# Chromatin remodeling factor lymphoid-specific helicase inhibits ferroptosis through lipid metabolic genes in lung cancer progression

**DOI:** 10.1186/s40880-017-0248-x

**Published:** 2017-10-16

**Authors:** Yiqun Jiang, Yuchen He, Shuang Liu, Yongguang Tao

**Affiliations:** 1 Key Laboratory of Carcinogenesis and Cancer Invasion, Ministry of Education, Xiangya Hospital, Central South University, Changsha, 410008 Hunan P. R. China; 2 Key Laboratory of Carcinogenesis, Ministry of Health, Cancer Research Institute, School of Basic Medicine, Central South University, Changsha, 410078 Hunan P. R. China; 3 Institute of Medical Sciences, Xiangya Hospital, Central South University, Changsha, 410008 Hunan P. R. China; 4 Department of Thoracic Surgery, Second Xiangya Hospital, Central South University, Changsha, 410011 Hunan P. R. China

Ferroptosis, a novel mode of non-apoptotic cell death, involves a metabolic dysfunction that results in the production of iron-dependent reactive oxygen species (ROS), an iron carrier protein (transferrin), intracellular metabolic process, and related regulators (e.g., p53 protein). Previous studies have linked ferroptosis with oncogenic *Ras* [1], and p53 tumor suppressor positively regulates ferroptosis by transcriptionally inhibiting the expression of the cysteine/glutamate antiporter, which is encoded by the *SLC7A11* gene in human [1, 2]. Whether other factors such as epigenetic factors are involved in the process remains less known.

Chromatin modifier lymphoid specific helicase (LSH) contributes to the malignant progression of nasopharyngeal carcinoma and glioma [3]. We recently indicated that LSH was shown to co-operate with partners, such as G9a, to drive cancer progression [4, 5]. However, the molecular mechanisms, particularly in lung cancer, are not well understood. Importantly, the impact of ferroptosis in cancer progression especially in chromatin remodeling is still far from fully understood. Based on the study reported in the article entitled “EGLN1/c-Myc induced lymphoid-specific helicase inhibits ferroptosis through lipid metabolic gene expression changes,” which was recently published in *Theranostics* by Jiang et al. [6], such an interplay between epigenetic controls in chromatin remodeling and ferroptosis has been addressed.

Using RNA sequencing and the gene ontology analysis, we first identified a significant enrichment in pathways that related to metabolic process and the Warburg effect [6]. Moreover, the link between LSH and metabolic genes prompted us to assess the expression of two groups of metabolic genes. The first group comprised glucose transporters (GLUTs), which were important in glucose transport, and the other group comprised fatty acid desaturases (FADSs), which were dependent on reduced nicotinamide adenine dinucleotide phosphate (NAPDH). We demonstrated that LSH contributes to lung cancer progression by directly up-regulating metabolic genes including stearoyl-CoA desaturase 1 (SCD1) and FADS2. LSH-mediated increases in metabolic gene expression may occur through a DNA methylation-independent mechanism rather than through chromatin regulation [4, 7]. Furthermore, our findings provided evidence for an interaction between LSH and WD repeat domain 76 (WDR76), which is a nuclear protein containing tandem copies of WD repeats (also known as WD40 or β-transducin repeats) that has unknown function in mammals. The LSH-dependent recruitment of WDR76 to the metabolic gene promoters and the subsequent chromatin modification that leads to metabolic gene activation links epigenetic regulation by LSH to up-regulation of the emerging metabolic genes.

The ferroptotic mode of programmed necrosis was recently discovered as an apoptosis-independent form of cell death in Ras-transformed cells; the *K*-*ras* mutant is common in lung cancer [8]. Ferroptotic death is morphologically, biochemically, and genetically distinct from apoptosis, necrosis (various forms), and autophagy. This process is characterized by an overwhelming, iron-dependent accumulation of lethal lipid ROS [1, 2]. We next demonstrated that LSH decreases the lipid ROS and iron concentrations, which supports an inhibitory role of LSH in ferroptosis [6]. We demonstrated that LSH is resistant to ferroptotic cell death in cancer cells after the treatment of erastin, a ferroptosis inducer, and inhibits ferroptosis by inhibiting the cysteine/glutamate antiporter system. RNA sequencing analysis results also showed that LSH is significantly associated with the metabolic process, indicating that LSH inhibits ferroptosis by affecting these metabolic genes [6]. Interestingly, antioxidant reagents, vitamin C, and aspirin do not affect the expression of LSH or mitochondria related genes [6]. Vitamin E is regarded as a highly efficient ferroptosis inhibitor. However, vitamin E did not affect LSH expression, indicating that types of cells and diseases might affect the efficiency of ferroptosis inhibitors. Lipid ROS and iron accumulation is a key characteristic of ferrotosis; we showed that both SCD1 and FADS2, which are linked with lipid metabolism, influenced ferroptosis by affecting the lipid ROS and iron levels [6]. Moreover, inducing ferroptosis including well-designed nanomedicines might provide a new insight to treat cancer.

The iron-dependent enzymes Egl nine homolog (EGLNs) catalyze hypoxia-inducible factor **(**HIF) prolyl hydroxylation, which leads to HIF-1α and HIF-2α degradation. HIF-1α regulates oxygen-dependent glucose and glutamine metabolism, playing a critical role in cancer progression [9]. In fact, EGLN1 inhibition causes accumulation of circulating metabolites [9]. Interestingly, some oncometabolites stimulate EGLN activity, which leads to diminished HIF levels. For example, high extracellular glutamate levels inhibit the xCT glutamate-cysteine antiporter (a glial transporter protein that exports substantial amounts of glutamate into the extracellular fluid) and thereby interfere with cysteine uptake, which results in decreased intracellular cysteine levels [9]. Decreased intracellular cysteine levels inhibit EGLN activity and stabilize HIF-1α [10]. We found previously that oncometabolites also activated LSH expression [4]; on the basis of this, our recent study found that EGLN1 up-regulated LSH expression by inhibiting HIF‐1α, which highlights HIF‐1α as a key repressor of LSH expression [6]. EGLN2 is essential for cell death and is a candidate driver of iron chelation-mediated inhibition of cell death. Interestingly, HIF-1α and c-Myc counteract each other. Our study found that c-Myc was recruited to the HIF-1α-binding site on the LSH promoter in the normoxic state [6].

**Fig. 1:**
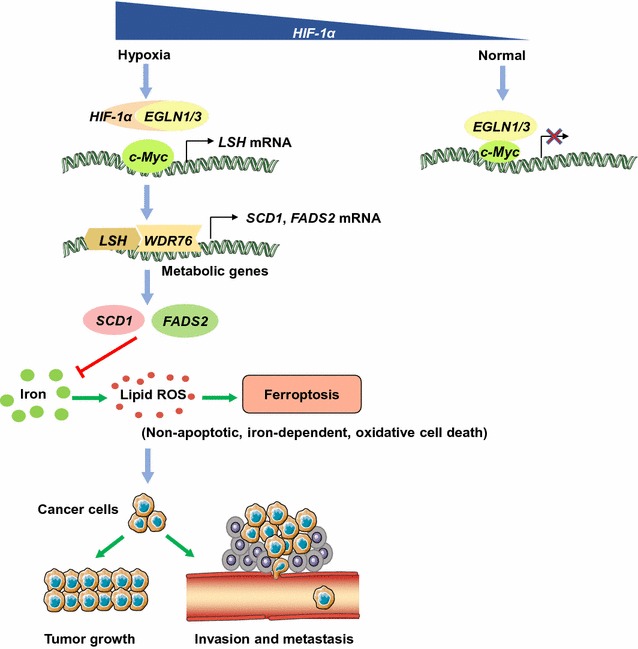
*LSH*-mediated inhibition of ferroptosis and enhancement of lung tumorigenesis. In this model, *LSH* acts as a novel inhibitor of ferroptosis by regulating several metabolism-related genes. *LSH* expression is up-regulated by *c*-*Myc*, which is enriched at the *LSH* promoter by the *EGLN1*-mediated repression of *HIF*-*1α*. The induced *LSH* interacts with *WDR76*, which, in turn, up-regulates the lipid metabolic genes including *SCD1* and *FADS2*. These metabolic genes inhibit the accumulation of lipid ROS and intracellular iron, which are required for ferroptosis, and inhibition of ferroptosis by LSH ultimately promotes cancer progression. *HIF-1α* hypoxia-inducible factor-1α, *EGLN1/3* Egl-9 family hypoxia-inducible factor 1/3, *LSH* lymphoid-specific helicase, *WDR76* WD repeat-containing protein 76, *SCD1* stearoyl-CoA desaturase 1, *FADS2* fatty acid desaturase 2, *ROS* reactive oxygen species

In summary, we demonstrated the crucial role of LSH in ferroptosis (Fig. 1) and considered LSH a potential therapeutic target for cancer treatment. Our findings demonstrate that ferroptosis is epigenetically regulated by LSH, which promotes lipid metabolic genes, including SCD1 and FADS2; both FADS2 and SCD1 link with the glutamate antiporter. Our results suggest that a preferential triggering of ferroptosis in cancer cells may serve as a viable therapeutic option.
